# CD4^+^CCR5^+^ T cells and CCL3+ mast cells are increased in the skin of patients with chronic spontaneous urticaria

**DOI:** 10.3389/fimmu.2024.1327040

**Published:** 2024-07-22

**Authors:** Raeda Mubariki, Reem Samara, Anna Maria Gimenez-Arnua, Marcus Maurer, Jacob Bejar, Elias Toubi, Zahava Vadasz

**Affiliations:** ^1^ The Unit of Proteomics and Flow Cytometry, Allergy and Clinical Immunology, Bnai-Zion Medical Center, Faculty of Medicine, Technion, Haifa, Israel; ^2^ Department of Pathology, Bnai-Zion Medical Center, Faculty of Medicine, Technion, Haifa, Israel; ^3^ Department of Dermatology, Hospital del Mar & Research Institute, Universitat Pompeu Fabra, Barcelona, Spain; ^4^ Institute of Allergology, Charité – Universitätsmedizin Berlin, corporate member of Freie Universität Berlin and Humboldt-Universität zu Berlin, Berlin, Germany; ^5^ Fraunhofer Institute for Translational Medicine and Pharmacology ITMP, Immunology and Allergology, Berlin, Germany

**Keywords:** CSU, T cells, mast cells, chemokines, wheals, angioedema

## Abstract

**Background:**

The proximity of activated T cells and mast cells in the lesional skin of patients with chronic spontaneous urticaria (CSU) is held to contribute to the development of wheals and angioedema. In a previous study, we demonstrated that increased IL-17 expression in T cells and mast cells in skin lesions of patients with CSU is associated with T/mast cell proximity, but the mechanisms that drive T cell/mast cell co-localization remain unknown.

**Objectives:**

To assess if chemokines expressed in lesional CSU skin contribute to T cell/mast cell proximity.

**Patients and methods:**

Biopsies from lesional CSU skin were compared to biopsies from healthy skin for expression of CCR5 and its ligand CCL3 by CD4^+^ T cells and mast cells, respectively.

**Results:**

Numbers of CCR5-positive CD4^+^ T cells in lesional CSU skin were significantly increased as compared to healthy normal skin (p < 0.0001). The number of mast cells expressing CCL3 (ligand for CCR5) in CSU skin was also increased (p < 0.0002) and significant association with T-cell close proximity (p < 0.0001) is noticed.

**Conclusions:**

The close proximity of T cells and mast cells in the skin of severe CSU may be driven, at least in part by increased CCR5 and CCL3 expression. Therapies that target CCL3 interaction with CCR5 should be assessed for their effects in CSU.

## 1 Introduction

Type I and type II autoimmunity are both involved in the pathogenesis of chronic spontaneous urticaria (CSU) (1, 2). As part of this, activated CD4^+^ T cells, namely, autoreactive T cells, were reported in many studies to be key players in CSU, both in the peripheral blood and in the lesional skin of CSU patients (3). Lesional CSU skin is characterized by perivascular non-necrotizing cellular infiltrate around small venules that consist mainly of CD4^+^ T cells, eosinophils, basophils, and neutrophils (4). The crosstalk between activated CD4^+^ T cells and mast cells was reported more than two decades ago, resulting into mast cell degranulation and the release of histamine and other proinflammatory mediators (5). During the process of this crosstalk, mast cells have been found to be activated in T-cell-mediated inflammation by remaining in close physical proximity to activated CD4^+^ T cells (6). In a recent study, we described for the first time the intense infiltration of IL-17A expressing CD4^+^ T cells in the skin of CSU patients in close proximity with IL-17A expressing mast cells, suggesting this to be one of the important mechanisms in the induction of mast cell degranulation in CSU (7). However, the mechanisms that drive T-cell infiltration and their co-localization with mast cells in the skin of CSU patients are yet to be better identified and characterized. Chemokines (both chemokine receptors and chemokine ligands) are small bound and secreted proteins, promoting the recruitment of activated immune cells into the cellular infiltrate of many inflammatory skin diseases including into lesional CSU skin, contributing to their differentiation and release of inflammatory cytokines (8, 9). Based on their molecular structure, chemokines are divided into ligands such as CCL and CXCL, which bind to their specific receptors, namely, to CCR or CXCR. In this respect, chemokine axis such as SDF-1/CXCR4 or CCL5/RANTES are reported to be potent chemo-attractants of proinflammatory immune cells and increased angiogenesis in inflamed skin tissues, such as chronic spontaneous urticaria and psoriasis (10, 11). Chemokines such as CXCL1/2, CXCL8, CCR5, and others are recently reported to be released from a wide spectrum of cells, including endothelial cells, activated T cells, and basophils in both peripheral blood and lesional skin of CSU patients resulting in the increased crosstalk between all these cells, thus contributing to the pathogenesis of CSU (12, 13). We suggest that many chemokine receptors on activated T cells and many chemokine ligands on mast cells are involved in this crosstalk between immune cells in the skin of CSU patients. In this study, we chose to analyze the expression of CCR5 (expressed on T cells) and CCL3 (expressed on mast cells) in order to establish for the first time their role in promoting the proximity of these cells in lesional skin of CSU patients.

## 2 Patients and methods

### 2.1 Patients

We analyzed 12 skin biopsies from lesional skin of severe CSU patients and compared them with eight biopsies from age- and sex-matched healthy individuals. Biopsies were taken following signing an informed consent and approved by the local Helsinki committee of Bnai-Zion Medical Center and the Charité—Universitätsmedizin Berlin. All CSU patients were unresponsive to high doses of anti-histamines, and short courses of steroids could not achieve sufficient remission. Biopsies were taken when patients did not use corticosteroids or cyclosporine A. Urticarial activity score (UAS7) values were available in all patients, ranging between 29 and 39 (mean ± 32) (see characteristics in **Table 1**). Biopsies from healthy individuals were taken from plastic surgeries, e.g., breast and abdomen. All biopsies were assessed for (1) the expression of CD4^+^ T cells and mast cells in lesional skin, in comparison with that in normal skin; (2) CCR5 expression on CD4^+^ T cells and the expression of CCL3 on mast cells; and (3) the proximity of CCL3-expressing mast cells with lymphocytes in the skin of CSU when compared to that in normal skin.

**Table 1:** Characteristics of CSU participants.

Participants characteristics (n = 12)		Mean
Sex	Female = 8, male = 4	
Age	28–62 years	±44 years
UAS7	34–40	±31 points
Disease duration	2–5 years	3 years
Comorbidities	Angioedema 4/12 Asthma 5/12 Autoimmunity 1/12	
Previous treatment	High doses of anti-histamines and short courses of steroids	
Current treatment	High doses of anti-histamines—before biological treatment	

### 2.2 Skin immunofluorescence

Lesional skin biopsies from severe CSU patients (n=12) and control skin biopsies (n=8) from healthy subjects were studied by double staining immunofluorescence. Briefly, formalin-fixed paraffin-embedded skin tissue sections were sliced serially into 5-µm slices by a microtome. The slides were deparaffinized by xylene and then rehydrated by absolute ethanol, 96% ethanol, 70% ethanol, and two short-distilled water washes. The antigen retrieval was done by cooker pressure in an EDTA solution (2 mM, and pH 8.0), blocked with 3% glycine for 1 h and then with 1% BSA for an additional hour (14). Primary antibodies, mouse monoclonal anti-human CCR5 (1:25, Abcam), rabbit polyclonal anti-human cKit (CD117) (1:100, Abcam), and rabbit polyclonal anti-human CD4 (1:300, Abcam) were incubated with the slides overnight. On the second day, slides were washed three times with 1× PBS and then incubated with secondary antibodies for 45 min. Slides were rewashed and stained with DAPI. The whole slide was scanned and quantified for the amount of CD4^+^ and cKit^+^ (CD117) and double stained for CD4^+^ T cells/CCR5 cells by using Image J software.

### 2.3 Skin immunohistochemistry

Tissues were fixed in 4% paraformaldehyde, processed routinely, and embedded in paraffin. Sections (3 μm) were mounted on positively charged slides. Hematoxylin/eosin staining was used for histological evaluation under a light microscope. Sequential sections were used for immunohistochemistry staining.

The immunostains were conducted on an automated stainer (Benchmark Ultra; Ventana Systems, Phoenix, AZ) (14). Following antigen retrieval in Tris-based buffer, double staining of CCL3 and CD117 (c-Kit) was performed. Antibodies used were rabbit anti-human CD117 polyclonal antibody (1:200, DAKO), rabbit anti-human CCL3 (1:500, Abcam). OptiVIEW DAB detection kit and UltraVIEW Alkaline phosphatase red detection kit (760-700 and 760-501, accordingly; Roche Diagnostics GmbH, Mannheim, Germany) were used according to manufacturer-recommended protocol, followed by hematoxylin counterstain for color development. Pictures of sections mounted for CCL3+CD117+ (c-Kit) were taken using a DP70 Olympus camera.

### 2.4 Statistical analysis

Results were presented as mean standard deviation (SD). The intensity of CD4^+^ T cells, mast cells, and chemokine expression was compared between CSU biopsies and healthy skin. Statistical significance was performed by Student’s t-test. Statistical significance was considered with a p-value ≤ 0.05).

### 2.5 Bioethical standards

Bioethical approval of the Charité—Universitätsmedizin Berlin (No. EA4/052/19) and that of the Bnai-Zion Medical Center, Israel (0133-18-BNZ) was obtained.

## 3 Results

### 3.1 CD4^+^ T cells and mast cells are increased in the CSU skin

**Figure 1:**
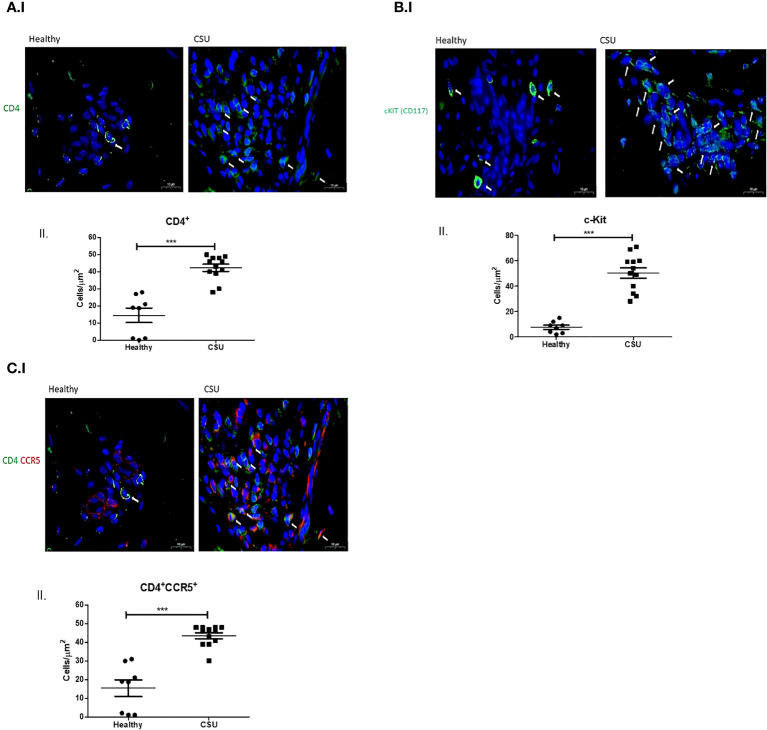
CD4^+^ T cells, mast cells, and CD4^+^CCR5^+^ in skin biopsies. **(A)** I. Normal skin shows small amount of CD4^+^ T cells while CSU skin shows numerous CD4^+^ T cells (in green, white arrows indicate CD4^+^ T cells). II. Quantitative analysis of number of CD4^+^ T cells in normal vs. CSU skin biopsies (p < 0.0001). **(B)** I. Normal skin shows small amount of mast cells, while CSU skin shows increased numbers of mast cells (in green, white arrows indicate mast cells). II. Quantitative analysis of number of mast cells in normal vs. CSU skin biopsies (p < 0.0001). **(C)** I. Double-staining immunofluorescence CD4^+^CCR5^+^ cells in CSU skin biopsies. Normal skin shows a small number of double-stained cells in comparison with CSU, in green and red, see white arrows. II. Quantitative analysis of number of double stained T cells in normal vs. CSU skin biopsies (p < 0.0001). Scale bar= 10 µm. *** statistically significant.

CD4^+^ T cells were markedly increased in the lesional skin of CSU patients, as assessed by quantitative histomorphometry. Specifically, CSU skin lesions had more than 2.8 fold higher numbers of CD4^+^ T cells as compared to the skin of healthy controls, i.e., 42.3 ± 2.1 vs. 14.7 ± 4.7 CD4^+^ T cells/µm^2^ (p < 0.0001, **Figure 1A**, I, II). Mast cell numbers, assessed by staining for CD117, were also substantially increased in the lesional skin of patients with CSU. As compared to healthy control skin, lesional CSU skin had >600% the number of mast cells, i.e., 50.5 ± 3.7 vs. 7.7 ± 2.4 mast cells/µm^2^ (p < 0.0001, **Figure 1B**, I, II).

### 3.2 CCR5-positive CD4^+^ T cells are increased in CSU skin

In lesional CSU skin, numbers of CCR5-positive CD4^+^ T cells were almost threefold higher as compared to healthy skin, i.e., 43.6 ± 1.6 vs. 15.4 ± 4.9 cells/µm^2^ (p< 0. 0001, **Figure 1C**, I, II).

### 3.3 The chemokine ligand CCL3 on mast cells is increased in CSU skin

**Figure 2:**
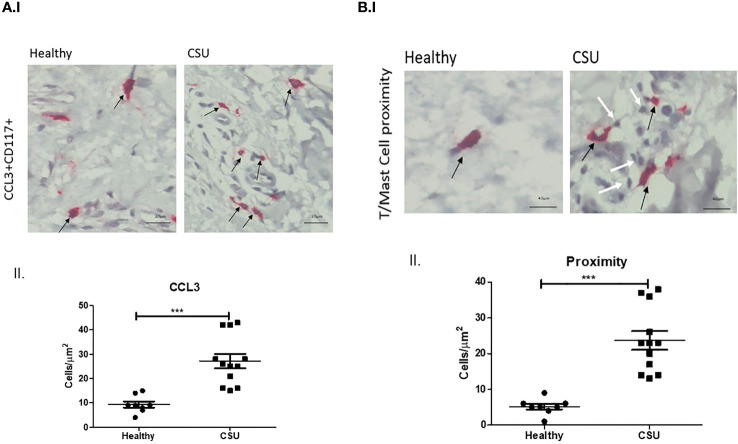
CCL3 expression on mast cells. **(A)** I. and II. Positive CCL3-stained mast cells in CSU skin biopsy. Black arrows denote mast cell that are positive for CCL3 staining; an increase in the CCL3-positive mast cell numbers is noted. ×200 magnification (p < 0.0002). Scale bar= 20 µm. **(B)** I. and II. CCL3-positive mast cells in CSU skin biopsy (black arrows), in proximity to CD4^+^ T cells (white arrows). ×400 magnification (p < 0.0001). Scale bar= 40 µm. *** statistically significant.

The numbers of mast cells that express CCL3, the ligand of CCR5, were markedly increased in lesional skin biopsies of CSU patient in comparison with normal skin biopsies, i.e., 27.1 ± 2.9 vs. 9.4 ± 1.4 cells/µm^2^ (p<0.0002) (**Figure 2A**, I, II). Most CCL3-positive mast cells, 23.4 ± 2.9 cells/µm^2^ (spindle-shaped nuclei with cytoplasmic granules) positioned in close proximity to T cells (round-shaped nuclei with small non-granular cytoplasm) (**Figure 2B**, I, II). The distance between T cells and mast cells CCL3+ was between 0.4 μm and 0.9μm in CSU skin biopsies comparing to 2.3–10.5 μm in healthy controls. Thus, a distance lower than 1 μm was considered to be in close proximity (see **Supplementary Figures**).

## 4 Discussion

IgE- and IgG-dependent autoimmunity is considered a major mechanism in the development of mast cell activation and degranulation in CSU skin. IgE-independent pathways are recently reported to play pivotal roles in the pathogenesis of mast cell activation (though of limited relevance). In this respect, human mast cells express several regulatory receptors such as toll-like receptors, Mas-related G-protein coupled receptor-X2 (MRGPRX2), and others. MRGPRX2 is expressed at high levels on skin mast cells and is activated by small molecule compounds during immune-mediated inflammation, leading to mast cell hypersensitivity reactions such as in CSU. This suggests that MRGPRX2 inhibitors may become a therapeutic strategy in allergic diseases (15). In addition, the crosstalk between infiltrating immune cells around small venules in deep dermis of CSU patients, namely, between T cells, mast cells, eosinophils, basophils, and neutrophils was previously shown to involve the presence of chemokines in the skin of atopic patients, thus becoming a highly relevant mechanism in mast cell degranulation in the pathogenesis of CSU (12). With this in mind, the proximity between activated CD4^+^ T cells and mast cells is highly appreciated to be a fundamental mechanism for the induction of mast cell degranulation. Y. Mekori was the first to characterize a novel mast cell activation pathway initiated by close physical contact with activated T cells. It was shown that mast cells were activated by the release of microparticles from activated CD4^+^ T cells involving MARK signaling pathway (16). In another study, microRNA-4443 derived by released microvesicles was shown to act as intercellular carriers, contributing to the downregulation of tyrosine phosphatase protein expression in mast cells, leading to their activation and degranulation (17). Later, we published our results where we demonstrated that increased IL-17 expression in both CD4^+^ T cells and mast cells were found to be in close proximity in lesional skin of CSU patients (7). In our current study, we clearly show for the first time that the expression of chemokine receptors and ligands on both CD4^+^ T cells and mast cells is significantly increased (**Figure 1C**, I, II, **Figure 2A**, I, II). In this respect, it is our additional finding that T cells are increasingly infiltrated in close proximity with CCL3 expressing mast cells (**Figure 2B**, I, II). In an early study, it was shown that following the binding of CCL3 to its CCR5 receptor on T cells, the phosphorylation and activation of Janus kinase 2 (JAK2) was observed, but this was not prevented by inhibition of JAK, indicating that this is only one of many other dependent signals (18). In another study, although not related to CSU, it was shown that the CCL3–CCR5 axis promotes cell migration and invasion of colon adenocarcinoma via Akt signaling pathway (19). Finally, increased expression of CCR5 and its ligand CCL3 was demonstrated in the tear film and ocular surface of patients with dry eye syndrome, especially in those with Sjogren’s syndrome (20). Our preliminary results in this study indicate clearly that CCR5–CCL3 axis is important in the crosstalk between mast cells and T cells in CSU lesional skin. This suggests that increased chemokine expression in CSU skin is of high relevance in attracting CD4^+^ T cells into the CSU skin and, by a crosstalk mechanism, may lead to mast cell activation and degranulation.

## 5 Study limitations

The limitations of our study are the relatively small number of available biopsies. This is because biopsies are not performed routinely in CSU patients, and they are rarely available for research purposes. In addition, functional tests are required in order to better establish the above-demonstrated observation. Additional studies may establish the idea that targeting this chemokine axis may become one of the optional therapeutic strategies in CSU (21).

## 6 Conclusions

Increased expression of CCR5 and its ligand CCL3 in T cells and mast cells in the skin of CSU patients may increase the proximity between these cells, which leads to disease worsening. Therefore, therapies that target CCL3 interaction with CCR5 should be assessed for their effects in CSU.

## Data availability statement

The original contributions presented in the study are included in the article/**Supplementary Material**. Further inquiries can be directed to the corresponding author.

## Ethics statement

The studies involving humans were approved by Bnai-Zion Local Helsinky Committee. The studies were conducted in accordance with the local legislation and institutional requirements. The ethics committee/institutional review board waived the requirement of written informed consent for participation from the participants or the participants’ legal guardians/next of kin because it is a retrospective study on skin biopsies.

## Author contributions

RM: Formal analysis, Investigation, Methodology, Software, Validation, Visualization, Writing – original draft. RS: Conceptualization, Data curation, Investigation, Methodology, Writing – review & editing. AG: Conceptualization, Data curation, Writing – review & editing. MM: Conceptualization, Data curation, Writing – review & editing. JB: Investigation, Methodology, Visualization, Writing – review & editing. ET: Conceptualization, Investigation, Methodology, Supervision, Writing – original draft, Writing – review & editing. ZV: Conceptualization, Data curation, Investigation, Methodology, Supervision, Writing – original draft, Writing – review & editing.
