# Supplementary figures and images for: CD4+CCR5+ T cells and CCL3+ mast cells are increased in the skin of patients with chronic spontaneous urticaria
Source: Front Immunol. 2024 Jul 22;15:1327040. doi: 10.3389/fimmu.2024.1327040 (PMC11298339; doi:10.3389/fimmu.2024.1327040)

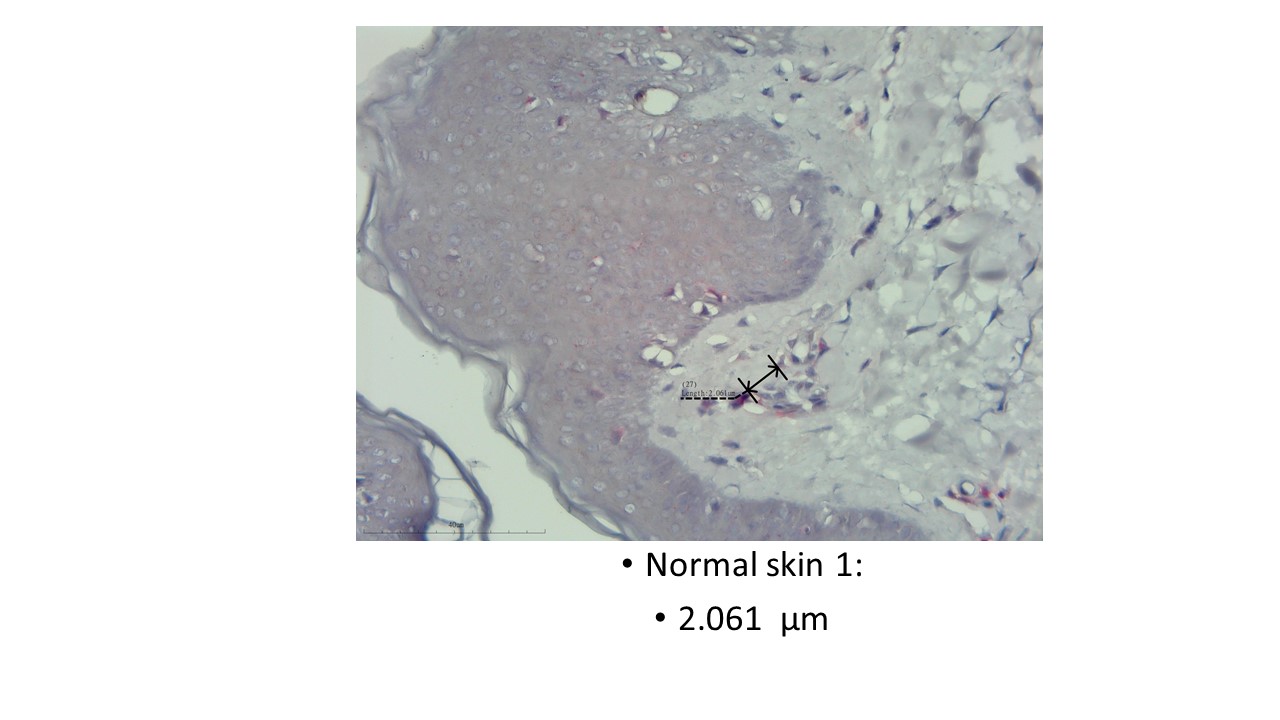

Supplement: Supplementary file 1 [file Image_1.jpeg]

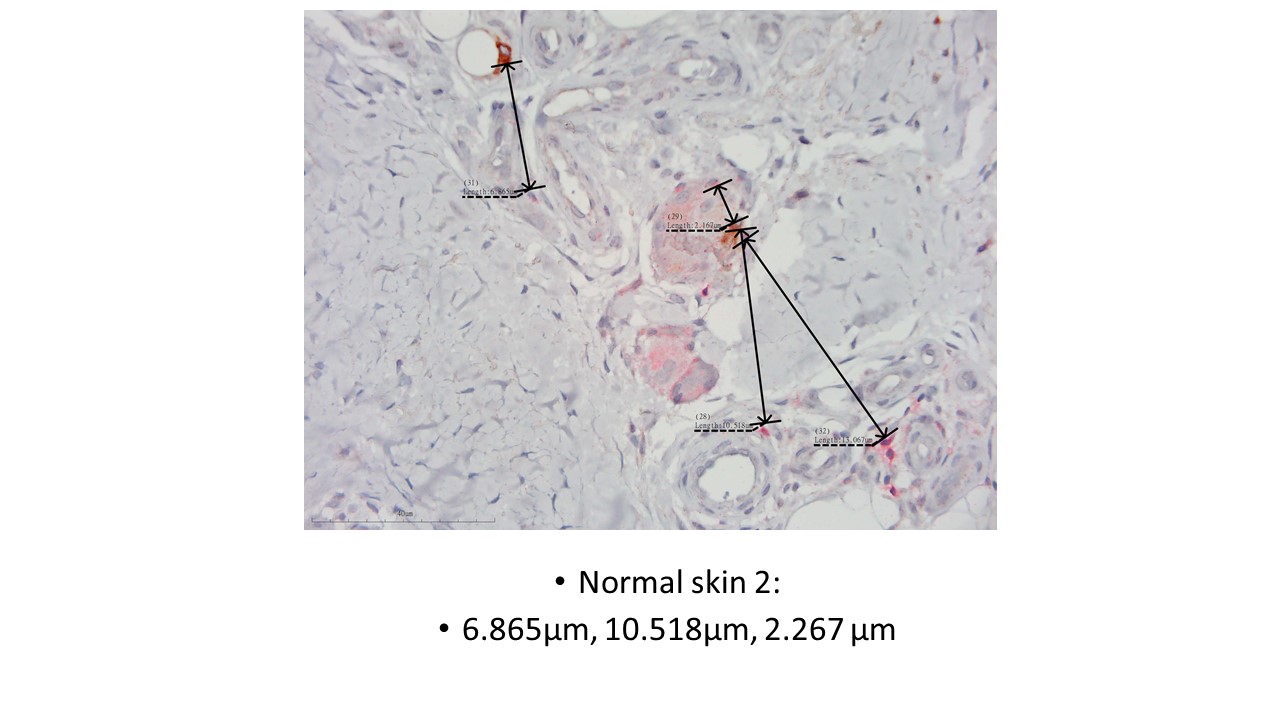

Supplement: Supplementary file 2 [file Image_2.jpeg]

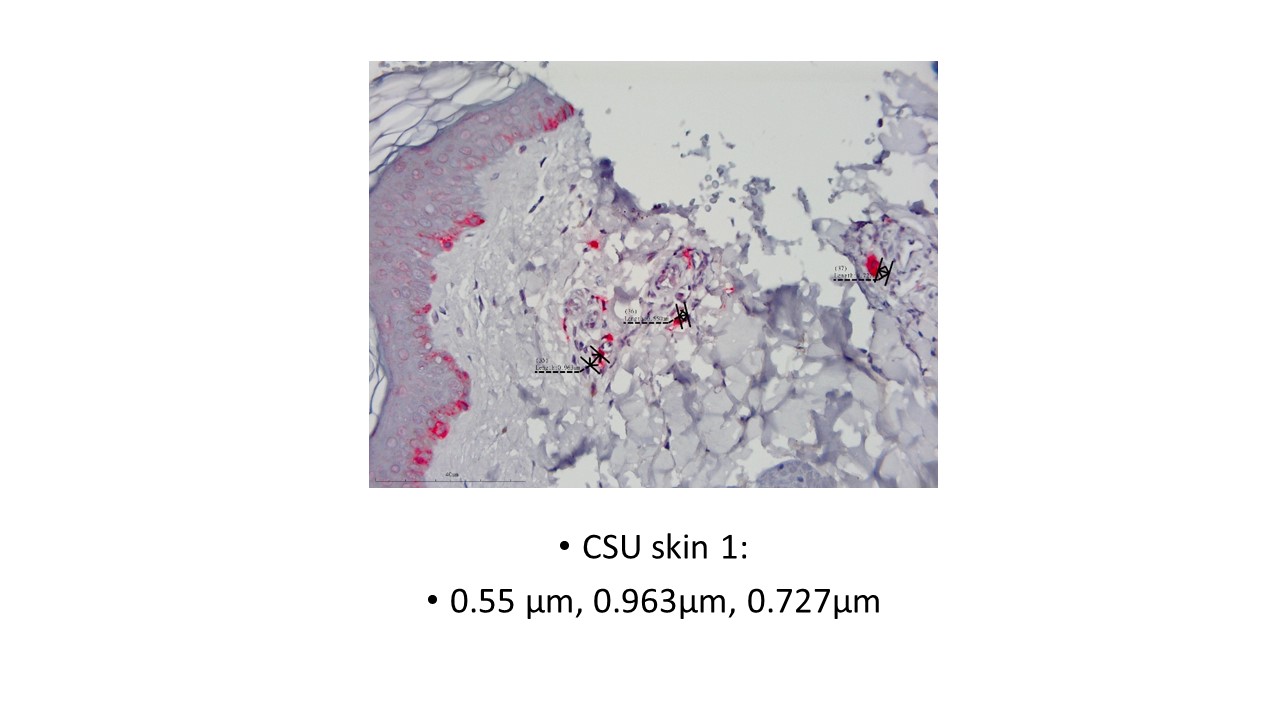

Supplement: Supplementary file 3 [file Image_3.jpeg]

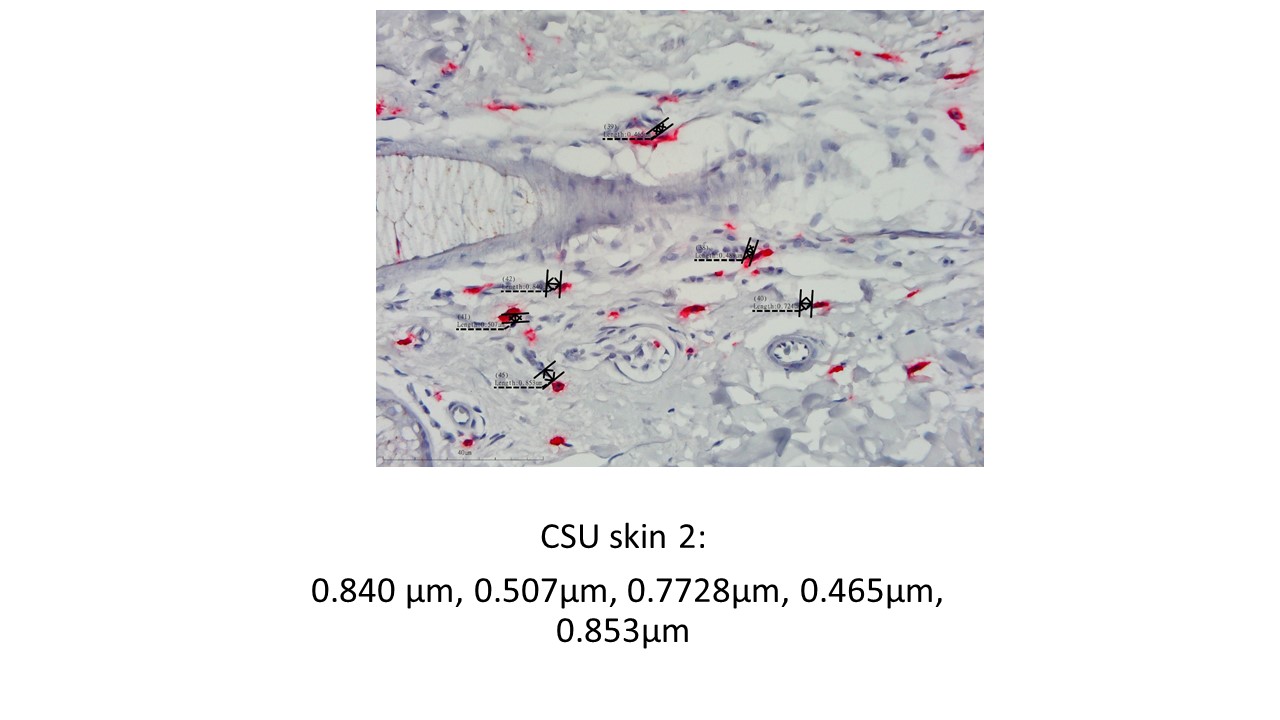

Supplement: Supplementary file 4 [file Image_4.jpeg]
